# Structural Insights of Electrical Aging in PZT Thin Films as Revealed by In Situ Biasing X-ray Diffraction

**DOI:** 10.3390/ma14164500

**Published:** 2021-08-11

**Authors:** Kien Nguyen, Ewen Bellec, Edoardo Zatterin, Gwenael Le Rhun, Patrice Gergaud, Nicolas Vaxelaire

**Affiliations:** 1 University Grenoble Alpes, CEA, Leti, F-38000 Grenoble, France; gwenael.le-rhun@cea.fr (G.L.R.); patrice.gergaud@cea.fr (P.G.); 2 ESRF, The European Synchrotron, 71 Avenue des Martyrs, CS40220, CEDEX 9, 38043 Grenoble, France; ewen.bellec@esrf.fr (E.B.); edoardo.zatterin@esrf.fr (E.Z.)

**Keywords:** in situ biasing X-ray diffraction, PZT thin film, MPB, ferroelectric domains, effective piezoelectric coefficient, electrical aging

## Abstract

Electrical aging in lead zirconate titanate (PbZr_x_Ti_1−x_O_3_) thin films has been intensively studied from a macroscopic perspective. However, structural origins and consequences of such degradation are less documented. In this study, we have used synchrotron radiation to evaluate the behavior of ferroelectric domains by X-ray diffraction (XRD). The sample was loaded with an AC triangular bias waveform between ±10 V with a number of cycle varying from one up to 10^8^. At each step of the aging procedure, XRD spectra had been collected in situ during the application of an electric field on a capacitor. The fine analysis of the (200) pseudo-cubic peak structure allows to separate the evolution of the volume of a/c tetragonal and rhombohedral domains along the electrical biasing. Throughout the aging, both intrinsic and extrinsic responses of tetra and rhombohedral domains are altered, the behavior depending on the observed phase. This methodology opens up new perspectives in the comprehension of the aging effect in ferroelectric thin film.

## 1. Introduction

Piezo/ferroelectric materials are widely used for many applications such as transducers [1], energy harvesters [2], sensors [3], RF filters [4], and memories [5]. Piezoelectricity is the ability of materials to generate electricity in response to a mechanical stress. Ferroelectric materials are part of the family of piezoelectric materials and are characterized by a spontaneous electric polarization below a certain temperature, the so-called Curie temperature. This polarization can be reversed by the application of an external electric field. Among all the materials, Pb(Zr_x,_ Ti_1−x_)O_3_ (PZT), or lead zirconate titanate, is one of the best known ferroelectric materials [6]. Of all the compositions of PZT, the one with a Zr/Ti atomic ratio close to 52/48, at the so-called morphotropic phase boundary (MPB), attracts a lot of attention due the strongly enhanced piezoelectric properties.

For technological applications, a key concern is the reliability of the piezo/ferroelectric properties of the materials with aging. Reliability is defined as the stability of the performance of the piezoelectric materials after being exposed to external factors. During the lifetime of piezoelectric materials, their performance could be affected by different factors such as the generation of oxygen vacancies, domain walls pinning, or defects of generation [7,8]. As a result, a number of studies have been conducted to investigate and improve the aging behavior of piezo/ferroelectric materials [9,10,11,12,13,14].

Piezoforce microscopy (PFM) [15,16] or double beam laser interferometer (DBLI) [17,18] are usually used to complement electrical testing diagnostics and they allow for the measurement of the effective piezoelectric coefficient *d*_33,*eff*_. However, these techniques present certain disadvantages. The correlation of *d*_33,*eff*_ with the *d*_15_ coefficient (PFM) or the occurrence of various forms of noise (thermal drift, fluctuation in the optical properties, and electrical and mechanical instabilities) in the case of DBLI could lead to some artifacts in the extraction of effective *d*_33,*eff*_.

**Figure 1:**
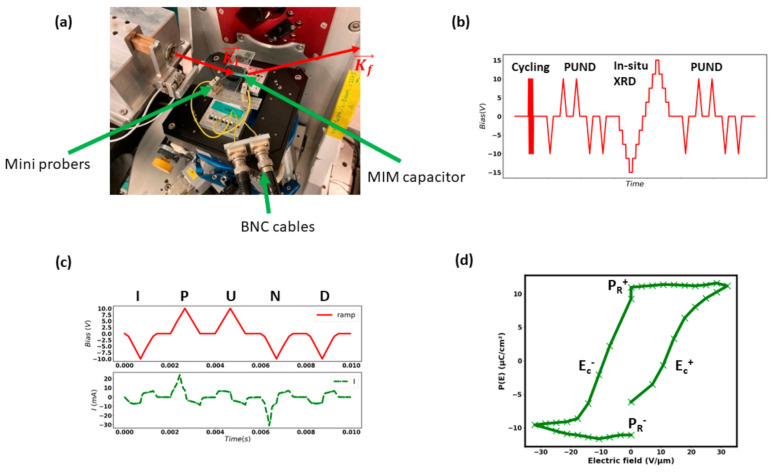
(**a**) The setup for the in situ biasing XRD experiment; (**b**) the experimental procedure; (**c**) the PUND waveform (red) and current response (green); and (**d**) the extract polarization loop from the PUND method.

In this study, the reliability of a prototypical PZT thin film at the morphotropic phase boundary (MPB) is investigated using an in situ biasing X-ray diffraction (XRD) procedure (as shown in Figure 1a,b), in addition to the ferroelectric measurement. The stability of the piezoelectricity is quantified through the evaluation of the effective piezoelectric coefficient *d*_33,*eff*_, extracted from the strain–electric field (S–E) curve. The term “effective” here signifies that the film is clamped to the substrate. As a result, the piezoelectric coefficient measured in our case will not be equivalent to that in the case of an unconstrained bulk sample [19]. In the case of in situ biasing XRD, as *d*_33,*eff*_ is directly deduced from the strain induced by the electric field at the crystallites level (few tenth of nanometers), which acts as strain gauge, any instabilities due to environmental factors and surface displacement can be prevented. In addition to a more accurate measurement of *d*_33,*eff*_, the main interest in this methodology lies in the fact that the domain types and their respective evolution under cycling can be extracted from the analysis of the Bragg peak fine structure. Such in situ biasing studies have formerly been developed for bulk piezoceramics [20,21,22] and have been recently extended to functional thin films [17,23,24,25].

In this study, we have adapted this in situ biasing XRD approach to address the structural consequence of the electrical aging of Pb(Zr_0.52_Ti_0.48_)O_3_ thin films grown by the sol–gel method. The impact of the AC electric field cycling on the structural properties of the sample will be first presented. Then, the evolution of the S–E curve and of the *d*_33,*eff*_ coefficient as a function of the number of cycles will be discussed. Finally, a correlation between the evolution of piezoelectric and ferroelectric aging of the sample will be proposed.

## 2. Materials and Methods

### 2.1. In Situ Biasing X-ray Diffraction Experiment

A prototypical PbZr_0.52_Ti_0.48_O_3_ thin film with a thickness of 0.5 µm has been studied. The sample was fabricated by a sol–gel route on TiO_2_ (20 nm)/SiO_2_ (500 nm)/Si (725 µm) substrate (further fabrication process and structural characterization information can be found in [26]). Pt bottom and Ru top electrodes 1 × 1 mm^2^ in size and 100 nm thick were used for electrical measurements.

Preliminary characterization by laboratory XRD using an energy of 8.05 keV indicated that the sample had a strong preferred (100) pseudo-cubic out of plane orientation.

The in situ X-ray diffraction experiment had been carried out at the ID01 beamline of the European Synchrotron Radiation Facility (ESRF) using a 100 × 100 µm^2^ beam monochromatized to an energy of 10.37 keV. The signal was recorded by a 2D detector (Maxipix© 516 × 516 pixels, for a pixel size of 55 µm). The measured Debye rings were then integrated to obtain a 1D spectra. A good peak intensity was obtained in only 1 s of counting time due to the high flux available on the beamline. The (200) pseudo-cubic peak of PZT was collected in a symmetrical coplanar configuration (i.e., the observed crystallites are oriented in the direction of electric field).

Two mini-micromanipulators were installed on the goniometer (Figure 1a) to perform the in situ biasing XRD experiment. The electric field was applied between ±20 V/µm by step of 2 V/µm. Considering the camera read-out, the in situ cycle frequency was performed at approximatively 10 mHz. In order to establish a stable electrical connection, the BNC contacts were fixed onto the piezo stage and then connected to the probers. These probers were put in contact with the bottom and top electrodes to apply the electrical bias as shown in Figure 1a.

### 2.2. Aging and Polarization Measurement

The aging procedure was performed with a triangular waveform between ± 10 V on the sample using an Arbitrary Functional Generator (AFG1000, Tektronix, Inc., Beaverton, OR, USA) at the frequency of 10 kHz for N = 1 to 10^8^ cycles. At each step of aging, the polarization was measured using a Source-Measure Unit (SMU) Keithley 2635 B (Keithley Instruments, Cleveland, OH, USA) controlled by a homemade Python script. Next, the in situ biasing XRD was performed. For each biasing, an XRD diagram is recorded. The control of the applied voltage and the duration of the pulse were done by a Python script, and the duration of the pulse matched exactly to the acquisition time of a single XRD scan. Finally, the measure of the polarization was conducted to follow the change in the polarization before and after the in situ biasing XRD. The whole procedure is summarized in Figure 1b.

The polarization was measured using the Positive-Up Negative-Down (PUND) method [27]. The typical waveform is schematized in Figure 1c. In order to understand this method, it is first necessary to remind the current density model of ferroelectric materials:

(1) $$J\left(t\right)=J_{switch}+J_{displacement\;}+J_{leakage}$$

where *J_switch_* is the current density due to the polarization switching, *J_displacement_* is the displacement current density, and *J_leakage_* is the leakage current density. The PUND technique consists of five bias pulses named I, P, U, N, and D. For the pulse I, the bias is applied to polarize the sample in a certain direction. After that, the pulse P is applied with the same magnitude but along the opposite direction to reverse the polarization. In this case, the current response of the sample consists of all the three contributions to the current density as mentioned above. Next, the pulse U is applied with the same magnitude and direction. However, as the polarization has already been reversed in the pulse P, after the pulse U, the current response will consist of only *J_displacement_* and *J_leakage_*. The same procedure is then applied with reversed direction for the N and D pulse. The current due to the application of the five pulses can be summarized by the following equations:

(2) I=initial pulse

(3) $$P=J_{switch}+J_{displacement}+J_{leakage}$$

(4) $$U=J_{displacement}+J_{leakage}$$

(5) $$N=J_{switch}+J_{displacement}+J_{leakage}$$

(6) $$D=J_{displacement}+J_{leakage}$$

By taking the current response of the P pulse minus the U pulse, and the N pulse minus the D pulse, the current density of *J_switch_* can be extracted. Finally, by integrating the *J_switch_* current as a function of time, the polarization loop P(E) can be plotted as shown in Figure 1d. The main features of the PUND method are illustrated in Figure 1c.

## 3. Results and Discussions

### 3.1. The Impact of Electric Field Cycling on the Structural Properties of PZT

**Figure 2:**
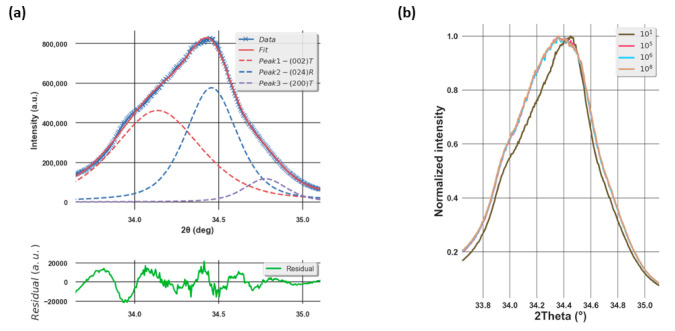
(**a**) XRD peak profile measured at the zero field and (**b**) the comparison of the peak profile from one up to 10^8^ electric cycles. (**b**) The XRD peak profile from the sample after one, 10^5^, 10^6^, and 10^8^ electric cycles. These are the peaks obtained at the zero field immediately after the AC cycling. It can be seen that after 10^8^ cycles, the intensity of the peak (002)T increases in comparison with that at one cycle at the expense of the (024)R reflection.

Due to the MPB composition of our sample, the (200) pseudo-cubic peak consists in fact of three peaks corresponding to the (002), the (200) peaks of the tetragonal phase (i.e., so-called a and c domain), and the (024) peak of the rhombohedral one [23]. As a result, in order to extract the evolution of each peak profile and deduce the structural properties, it is necessary to do a three-peak fitting. In our case, this was achieved using the least-square minimization (Lmfit) Python package on a pseudo-Voigt peak shape function as shown in Figure 2a.

**Figure 3:**
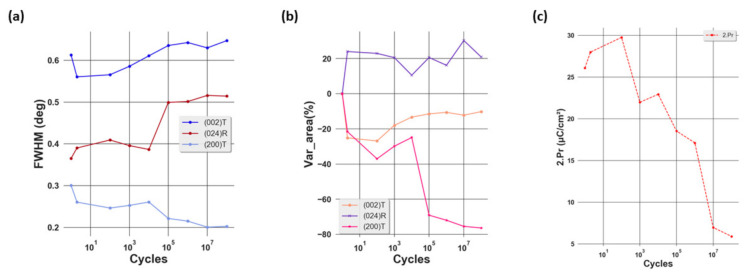
Evolution of (**a**) FWHM, (**b**) surface area of the XRD peak of different phases, and (**c**) the total remanent *2.P_R_* (*2.P_R_* = |*P_R_*^+^| + |*P_R_*^−^|) as a function of electric cycling.

After conducting the fitting on the XRD peak profiles, we are able to deduce the full width half maximum (FWHM), the position, and the surface area of the peak. While the first parameter gives an idea of the crystallite size, the third illustrates the volume of different crystallographic phases in the direction of reflection. Even though no significant evolution of the peak position with respect to the number of cycles was observed (not shown here), remarkable changes of the FWHM and surface area of the peak can be seen as shown in Figure 3a,b. After the first cycle, a drop in the FWHM of (002)T and (200)T phases, which corresponds to an increase in the crystallite size, was observed. This could be due to the elimination of the defects in the lattice. In ferroelectric materials, the accumulation of defects might affect the domain size and domain wall motion [28]. As a result, a decrease in the number of defects would lead to a larger crystallite size, which could be accompanied by a decrease in the FHWM. Simultaneously, an increase in the FWHM of the (024)R phase, corresponding to a decrease in the crystallite size, is observed. After this wake-up phase, while the FWHM of the (002)T and (024)R phases increase, corresponding to a decrease in the crystallite size, that of the (200)T phase decreases, thus indicating an increase in the crystallite size. Considering the surface area of the peak, after the first cycle a strong increase in that of the (024)R phase was obtained in addition to the decrease for the tetragonal phases, showing that there is a phase transformation from tetragonal to rhombohedral. After that, while the surface area of (024)R varies slightly, a sharp decrease was seen for the (200)T phase from two up to 10^8^ cycles, illustrating a decrease in the volume of this phase. Considering the (002)T phase, it can be seen that its volume increases gradually after 10^2^ cycles. A direct correlation to the total remanent *2.P_R_ which* decreases from this point is reported in Figure 3c.

### 3.2. Evolution of Piezoelectric Coefficient d_33,eff_ during Aging

**Figure 4:**
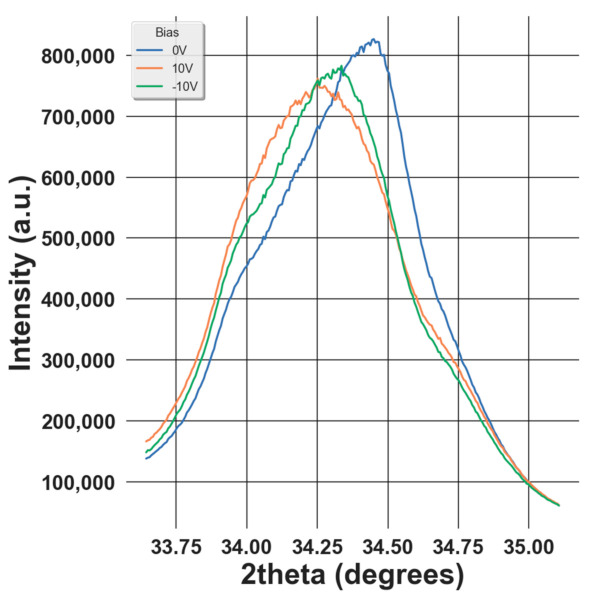
Variation XRD diagram as a function of the applied bias.

A piezoelectric material generates the electric field under applied stress and deforms under an electric field. The later phenomenon can be described from a microscopic point of view as the variation of the atomic d-spacing under an applied electric field. This deformation can be visualized by the shift of the XRD peak position (as shown in Figure 4) as the d-spacing is related to the peak position 2 *θ* through Bragg’s law:

(7) λ=2dsinθ

where λ is the wavelength of the incident beam, *d* is the d-spacing of an hkl reflection, and *θ* is the Bragg angle.

For studying the reliability of the piezoelectric properties in the PZT sample, the effective longitudinal piezoelectric coefficient *d*_33,*eff*_ is extracted from the S–E curves of the average strain after field cycling. In this case, the average strain is deduced from the center of gravity of the three peaks obtained at different electric fields which can be calculated by the following formula:

(8) $$\mathrm{Strain}\_\mathrm{avg}=\frac{Iint_{002}\varepsilon_{002}+Iint_{024}\varepsilon_{024}+Iint_{200}\varepsilon_{200}}{Iint_{002}+Iint_{024}+Iint_{200}}$$

where *Iint_hkl_* is the surface area and $\varepsilon_{hkl}$ is the strain for a specific atomic plane (hkl) that can be obtained from:

(9) $$\varepsilon_{hkl}=\frac{d_{hkl}-d_{hkl}^{0}}{d_{hkl}^{0}}$$

where $d_{hkl}$ and $d_{hkl}^{0}$ are the d-spacing of the (*hkl*) plane with and without an electric field, respectively [23]. As a result, a slight increase can also be observed for the strain, indicating an extension of the lattice.

**Figure 5:**
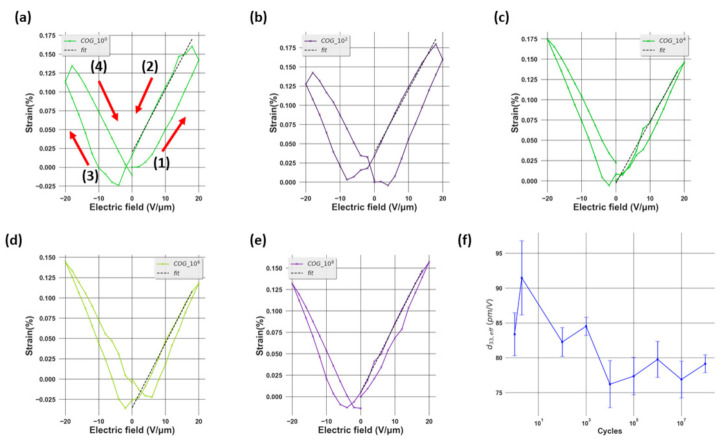
(**a**–**e**) The S–E curve at different numbers of electric field cycles with the first-order fitting for *d*_33_ calculation and (**f**) the variation of *d*_33,*eff*_ as a function of the number of electric field cycles.

By definition, the effective piezoelectric coefficient *d*_33,*eff*_ can be deduced from the following formula:

(10) $$d_{33,eff}={\left(\frac{\partial S_{3}}{\partial E_{3}}\right)}_{T}$$

where *S*_3_ and *E*_3_ refer to the strain and electric field along the out-of-plane orientation, and *T* is the stress. As a result, *d*_33,*eff*_ can be calculated by fitting the butterfly S–E curve with a first-order polynomial as shown in Figure 5a–e.

Looking at the Figure 5a–e, the first phenomenon observed is an increase in the asymmetry of the S–E loop and the collapse of the right wing of the butterfly curve. This can be explained by the existence of an internal bias and the domain-pinning effect [29]. The first effect leads to the asymmetry of the effective bias field in the sample, which is the superposition of the internal bias and the applied bias field. Considering the second factor, it is first necessary to recall that, under an external electric field, the strain in ferroelectric materials is generated by two main mechanisms: intrinsic and extrinsic. While the first stems from the linear piezoelectric effect, the second originates from the domain-switching effect [17]. In our study, the former can be characterized by the shifting of the XRD peak while the latter is illustrated by the increase of the intensity of the (002)T peak at the expense of the intensity of the (200)T phase under an applied electric field as shown in Figure 4, which corresponds to the non-180° domain-switching effect. Due to the domain-pinning effect, the domain walls are pinned so that the domains remain in the preferred polarization orientation even if under an external electric field. As a result, most of the strain is generated from the intrinsic mechanism. Furthermore, it can be seen that the maximum strain varies with the number of electric field cycles from one up to 10^8^ cycles. After 10^2^ cycles, the maximum strain increases from 0.16% to 0.18%. This could be understood as a result of the wake-up effect, which is evidenced from the increase of the remnant (*2.P_R_*) as illustrated in Figure 3c. During this cycling range, the domains are de-pinned. Thus, the amount of switched domains due to the non-180° switching mechanism increases, leading to higher strain. From 10^2^ up to 10^8^ cycles, the maximum strain decreases from 0.18 to approximately 0.16% and this could be explained as a result of the fatigue state, demonstrated by the decrease of *2.P_R_* from 10^2^ cycles as shown in Figure 3c. In this state, the pinned domains are more difficult to switch, thus decreasing the strain. Figure 5f shows the variation of *d*_33,*eff*_ as a function of the number of aging cycles. We can see that after two cycles, this quantity increases from around 87 to 96 pm/V. This could be due to the de-pinning effect of the domains, leading to larger non-180° domain switching. After that, *d*_33,*eff*_ decreases gradually to approximately 80 pm/V at 10^8^ cycles. This diminution could be attributed to the reduction of non-180° domain switching, which stems from the domain-pinning effect in the fatigued sample [30].

### 3.3. Evolution of Domain Behavior during Aging

**Figure 6:**
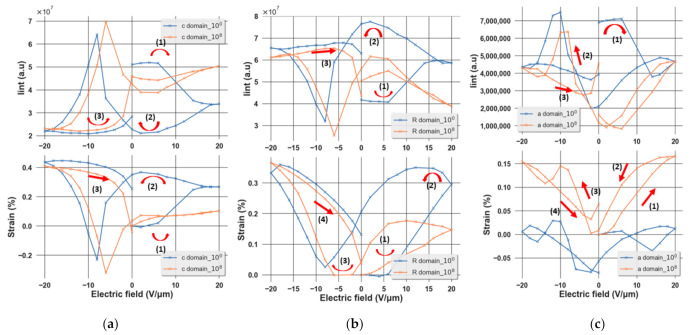
Variation of the peak area and strain of (**a**) the (002)T, (**b**) (024)R, and (**c**) (200)T phases as a function of an external electric field.

A butterfly shape of the peak area evolution and strain as a function of an applied electric field is obtained as shown in Figure 6a–c, indicating the ferroelectricity of the sample. It can be seen that as the applied electric field is increased, an increase in the area of the rhombohedral peak is observed, indicating the increment in the volume of this phase. Conversely, at a high electric field, the area of the (200) peak and (002) peak decreases, meaning that the volume of these phases decreases. From those two observations, it can be concluded that there is a phase transformation from tetragonal to rhombohedral in this sample under an external electric field, which is expected at the MPB composition of the sample [17,21]. Furthermore, after 10^8^ cycles, we observed the shifting of the butterfly loops towards the positive side, which could be understood as a result of the domain-pinning effect in the fatigue state of the film. Due to this effect, a higher electric field is needed to switch the domains. As a result, a collapse of the butterfly curve is observed, indicating that the mechanism of strain generation is dominated by the intrinsic effect.

## 4. Conclusions

In this study, the structural transformation and evolution of the longitudinal piezoelectric coefficient *d*_33,*eff*_ of PZT at the MPB composition have been investigated thanks to an in situ biasing X-ray diffraction methodology performed at a synchrotron light source. By refining the peak shape and position using a least-square minimization method based on a pseudo-Voigt peak shape model, we have been able to extract the evolution of the intensity, position, and FWHM of three overlapped peaks corresponding to the three domain types present in our film: (002)T, (024)R, and (200)T. Through the analysis of the first XRD peak after the electric field cycling (at the zero field), we have been able to investigate the impact of AC cycling on the crystallographic structure. Results show that there is a transformation from rhombohedral to tetragonal domains after 10^8^ aging cycles. Furthermore, an increase in the volume of the (002)T phase has been observed after 10^2^ aging cycles, which corresponds to the remnant fatigue point. By in situ biasing XRD, the S–E curves have been obtained at different numbers of cycles and have shown a diminution of the maximum strain when increasing the aging cycle. From this strain measurement, the *d*_33,*eff*_ coefficient was calculated; it increased after a short wake-up before decreasing with a trend similar to that observed for the *2.P_R_* as a function of the number of aging cycles. Both of these phenomena could be due to the domain-pinning effect in the fatigued sample, leading to the reduction of non-180° domain switching. Furthermore, when applying a DC electric field during in situ biasing XRD, we observed a transformation from the tetragonal to rhombohedral phase at high electric fields. Finally, by comparing the butterfly curves of strain and peak area versus electric field, it can be seen that after 10^8^ cycles, these curves collapse, indicating the degradation of the domain-switching effect in the fatigued state.

## Data Availability

The data presented in this study are available on request from the corresponding author. The data are not publicly available due to confidentiality.
